# Thousands of Lesions in Disseminated Cysticercosis

**DOI:** 10.4269/ajtmh.2011.11-0158

**Published:** 2011-10-01

**Authors:** Rajesh Verma, Pawan Sharma, Navdeep Khurana

**Affiliations:** Neurology, Chhatrapati Sahuji Maharaj Medical University, Lucknow, UP, India

Human cysticercosis is an important cause of seizures in the developing world. A small subset of patients acquires heavy infections with large numbers of lesions disseminated throughout the brain and skeletal muscle.^1^ Manifestations of heavy multiple cysticercotic syndromes include cysticercal encephalitis or signs and symptoms related to location. A 30-year-old man presented with intermittent partial complex seizures and headache for the previous 2 years. Examination revealed widespread small pea-sized nodules throughout the body, including the tongue (Figure 1). Calf hypertrophy was present (Figure 2). Higher mental function, cranial nerves, and motor, sensory, and reflexes were normal. An enzyme-linked immunsorbent assay for *Taenia solium* antibody was positive at high titers. A T2 sagittal magnetic resonance image of the brain showed numerous circumscribed lesions with visible scolices and involving adjacent soft tissues (Figure 3). Cysticercosis is caused by the cestode larvae of the pork tapeworm *T. solium*. Disseminated cysticercosis has the potential to cause severe clinical manifestations.^2^

**Figure 1.**
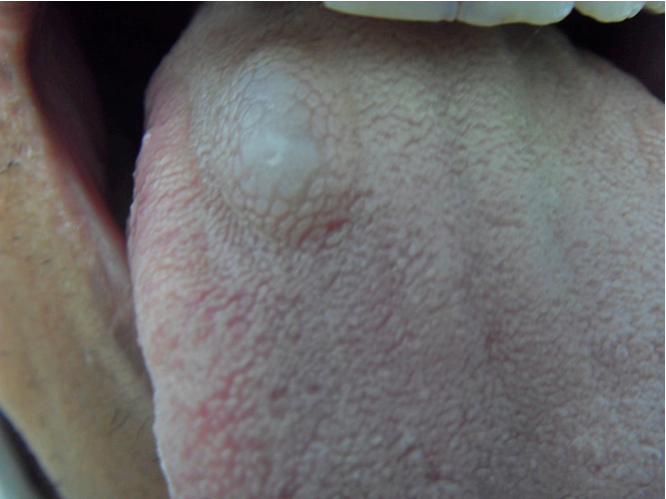
Tongue of the patient demonstrating larval cyst.

**Figure 2.**
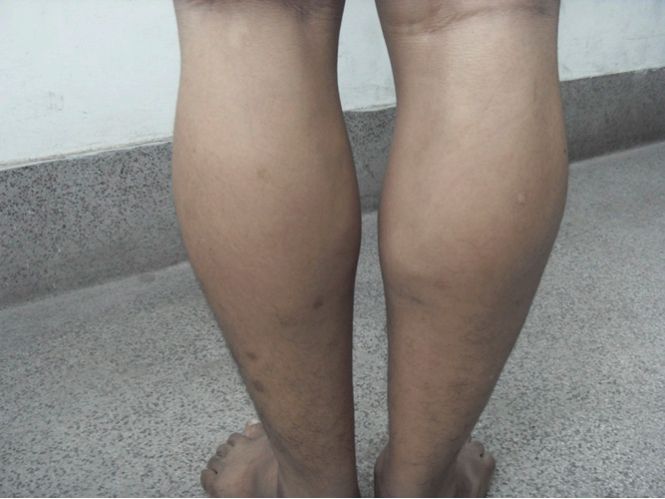
Photograph of the patient revealing calf hypertrophy.

**Figure 3.**
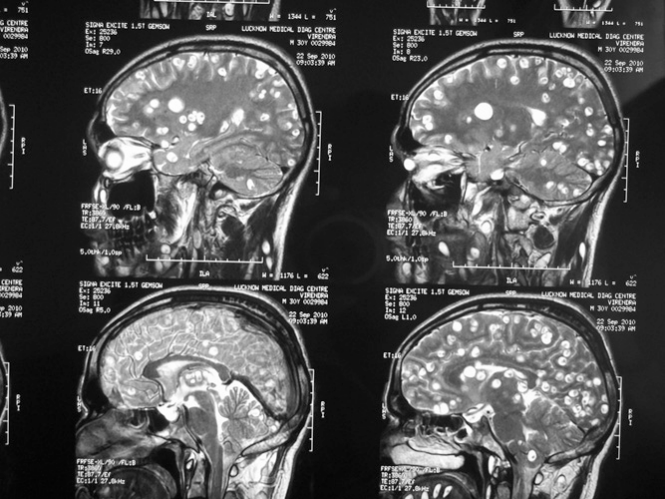
Magnetic resonance imaging of the cranium, T2 weighted sagittal image showing multiple well-circumscribed cysts with presence of scolex.

## References

[1] Pushker N, Bajaj MS, Balasubramanya R (2005). Disseminated cysticercosis involving orbit, brain and subcutaneous tissue. J Infect.

[2] Kumar A, Goenka AH, Choudhary A, Sahu JK, Gulati S (2010). Disseminated cysticercosis in a child: whole-body MR diagnosis with the use of parallel imaging. Pediatr Radiol.

